# Molecular mechanism of Epicedium treatment for depression based on network pharmacology and molecular docking technology

**DOI:** 10.1186/s12906-021-03389-w

**Published:** 2021-09-03

**Authors:** Yankai Dong, Bo Tao, Xing Xue, Caixia Feng, Yating Ren, Hengyu Ma, Junli Zhang, Yufang Si, Sisi Zhang, Si Liu, Hui Li, Jiahao Zhou, Ge Li, Zhifei Wang, Juanping Xie, Zhongliang Zhu

**Affiliations:** 1 Key Laboratory of Resource Biology and Biotechnology in Western China, Ministry of Education, College of Life Sciences, Institute of Maternal and Infant health, Northwest University, Xi’an, 710069 Shanxi Province China; 2 Department of Orthopaedic, Tianjin Medical University General Hospital, Anshan Road No.154, Tianjin, 300052 Heping District China; 3 Department of Neonatology, The First Affiliated Hospital of Medical College,Xi’an Jiaotong University, Xi’an, 710069 Shanxi Province China; 4 Qinba Chinese Medicine Resources R&D Center, School of Medicine, Ankang University, Ankang, 710069 Shanxi Province China

**Keywords:** Epimedium, depression, network pharmacology, molecular docking, pathway analysis

## Abstract

**Background:**

Increasing attention has been paid to the effect of *Epimedium* on the nervous system, particularly anti-depression function. In the present study, we applied network pharmacology to introduce a testable hypothesis on the multi-target mechanisms of *Epicedium* against depression.

**Methods:**

By reconstructing the network of protein–protein interaction and drug–component–target, we predicted the key protein targets of *Epicedium* for the treatment of depression. Then, through molecular docking, the interaction of the main active components of *Epicedium* and predicted candidate targets were verified.

**Results:**

Nineteen active compounds were selected from *Epicedium*. There were 200 targets associated with *Epicedium* and 537 targets related to depression. The key targets of *Epicedium* for treating depression were IL6, VEGFA, AKT1, and EGF. According to gene ontology functional enrichment analysis, 22 items of biological process (BP), 13 items of cell composition (CC) and 9 items of molecular function (MF) were obtained. A total of 56 signaling pathways (P < 0.05) were identified by Kyoto Encyclopedia of Genes and Genomes analysis, mainly involving depression-related pathways such as dopaminergic synapse, TNF signaling pathway, and prolactin signaling pathway. The results of molecular docking showed that the most important activity components, including luteoklin, quercetin and kaempferol, were well combined with the key targets.

**Conclusions:**

Luteoklin, quercetin, kaempferol and other active compounds in *Epicedium* can regulate multiple signaling pathways and targets such as IL6, AKT1, and EGF, therefore playing therapeutic roles in depression.

**Supplementary Information:**

The online version contains supplementary material available at 10.1186/s12906-021-03389-w.

## Background

Depression is a common chronic and highly disabling disorder with high level of treatment resistance [1]. Depression has affected more than 350 million people worldwide [2–4], and it is characterized by weight loss, low energy, loss of appetite, and insomnia [5, 6]. In recent years, most antidepressants have become NMDA receptor antagonists, which can produce antidepressant effects quickly, but these drugs are not used as first-line antidepressants because of their side effects, such as sensory agitation, cognitive impairment, addiction and hallucination [7, 8]. In addition, the pathogenesis of depression has been partly elucidated based molecular and genetic studies, but the potential mechanism of depression needs to be determined [9].

The primary task of prevention and treatment of depression is the development of new antidepressant therapeutic targets and therapeutic drugs with short onset latency and less side effects. Considering the improved safety and fewer side effects, traditional Chinese medicines (TCMs) performs an important role in the prevention and treatment of diseases [10], and have increasing attention among scientists worldwide. TCMs have multiple ingredients, targets and ways to be effective [11]. As a Chinese herbal medicine, *Epicedium* is well-known for wide-ranging effects, such as treatment of cancer, cardiovascular diseases, and immune suppression [12]. The extract of *Epicedium* and its component icariin can effectively promote the regeneration of peripheral nerve and improve the damaged nerve function [13]. *Epicedium* ethanol extraction exerts an anti-inflammatory effect by inhibiting the production of tumor necrosis factor-α, interleukin (IL)-1β, and IL-6 in lipopolysaccharide(LPS)-induced peritonitis [14]. Icariin can ameliorate depressive behavior of male offspring with prenatal stress [15]. In addition, *Epicedium* is rich in chemical composition, including flavonoids, lignans, and polysaccharides [12]. However, the therapeutic mechanism of *Epicedium* on depression is not clear.

With the rapid progress of bioinformatics, systems biology, and poly-pharmacology, network-based drug discovery has become a promising approach for the development of effective drugs. In 2007, Hopkins et al. first proposed the concept “network pharmacology”. This method analyzes the intervention of drugs and potential treated targets of diseases based on system biology [16, 17]. Network pharmacology highlights a paradigm shift from the current “one target, one drug” strategy to a novel version of the “network target, multi-component” strategy [18]. In TCM research, it is widely used because of the integrity and system consistent with the TCM prescription’s principles [19, 20]. As a computer-aided drug design method that depending on the interaction and affinity between the target and active compound, molecular docking has been widely used in the material basis of TCMs [21].

In the present study, we applied network pharmacology and molecular docking to clarify the mechanism of *Epicedium* in the treatment of depression. We systematically analyzed the active ingredients, potential targets, pathways and networks affected by *Epicedium* for the treatment of depression. We also performed molecular docking studies to predict the interactions that allow important compounds to bind to its predicted targets. Our results may help clarify how *Epicedium* can be effective against depression and facilitate the development of novel drugs.

## Methods

### Prediction of target genes associated with depression

Genes related to depression were obtained from the National Center for Biotechnology Information database (https://www.ncbi.nlm.nih.gov/) through searching “depression”. After filtering with the term “Homo sapiens", 537 genes associated with depression were identified in the Gene Bank database.

### Collection of component targets of *Epicedium*

The compounds and targets of *Epicedium* were mainly collected from TCMSP, a natural product database for Chinese herbal medicines (http://ibts.hkbu.edu.hk/LSP/tcmsp.php,updated on May 31, 2014 )[22]. In clinical treatment, TCMs are often used via oral administration. Oral bioavailability (OB)] [23] and drug-likeness (DL )[24], which are ADME-related models, mainly affect the absorption of drugs by the gastrointestinal tract. Therefore, we screened bioactive components under the standards of OB≥30% and DL≥0.18 [25], and obtained the related targets of each components. The common targets of *Epicedium* and the depression related genes were intersected with the Venn map. The intersected genes were the target genes of *Epicedium* for the treatment of depression.

### Protein–protein Interaction (PPI) Data

String database (https://string-db.org/cgi/input.pl) [26] contains known and predicted protein-protein interaction. A large number of PPIs were collected involving 9643763 proteins and 1380838440 interactions, including data obtained from experimental detection and bioinformatics prediction. The intersected genes were imported into the string database. The species was defined as “Homo sapiens”. The PPI data was obtained. The results were saved in TSV format. The information of node1, node2 and combined score in the file were retained. The interaction network was drawn and the network was analyzed by cytoscape.

### Network Construction

The active components of *Epicedium* and intersected genes were imported into Cytoscape version 3.6.0 to construct the compound-target network of *Epicedium*.

### Gene Ontology (GO) and Pathway Analysis

We used the Database for Annotation, Visualization, and Integrated Discovery [27] (DAVID, https://david.ncifcrf. gov/, v6.8) database for GO enrichment analysis and accomplished pathway enrichment analysis by using Kyoto Encyclopedia of Genes and Genomes [28](KEGG, http://www.kegg.jp/). Biological information annotation database (David, https://david. ncifcrf.gov/ , version 6.8) provides a systematic and comprehensive annotation information on the biological functions of large-scale genes or proteins, and it can determine the most considerably enriched biological annotation. The insected genes were imported into the David database. The selected identifier was set to official gene symbol. The list type was set to gene list, and the species was limited to “Homo sapiens”. Go analysis and KEGG pathway analysis were performed on the insected genes.

### Molecular docking

The crystal structures of the candidate protein targets of *Epicedium* were downloaded from the RCSB Protein Data Bank (http://www.pdb.org/) and modified using the Autodock tools 1.5.6 software. The four targets were IL6 (PDB ID: 5fuc )[29], VEGFA (PDB ID: 6d3o; https://www1.rcsb.org/structure/6d3o), AKT1(PDB ID: 6s9x) [30], and EGF (PDB ID:1jl9) )[31]; these targets include ligand and water removal, hydrogen addition, and amino acid optimization and patching. The files were saved in pdbqt format. ChemBioDraw 3D was used to create the 3D chemical structures and minimize their energy. Results were saved in MOL.2 format. The compounds were imported into Autodock tools 1.5.6, and all flexible keys were rotatable by default and saved in pdbqt format, as docking ligand. Autodock Vina 1.1.2 was used for docking, while Discovery Studio 3.5 was used to visualize the docking results.

## Results

### Active compounds of *Epicedium*

**Fig. 1:**
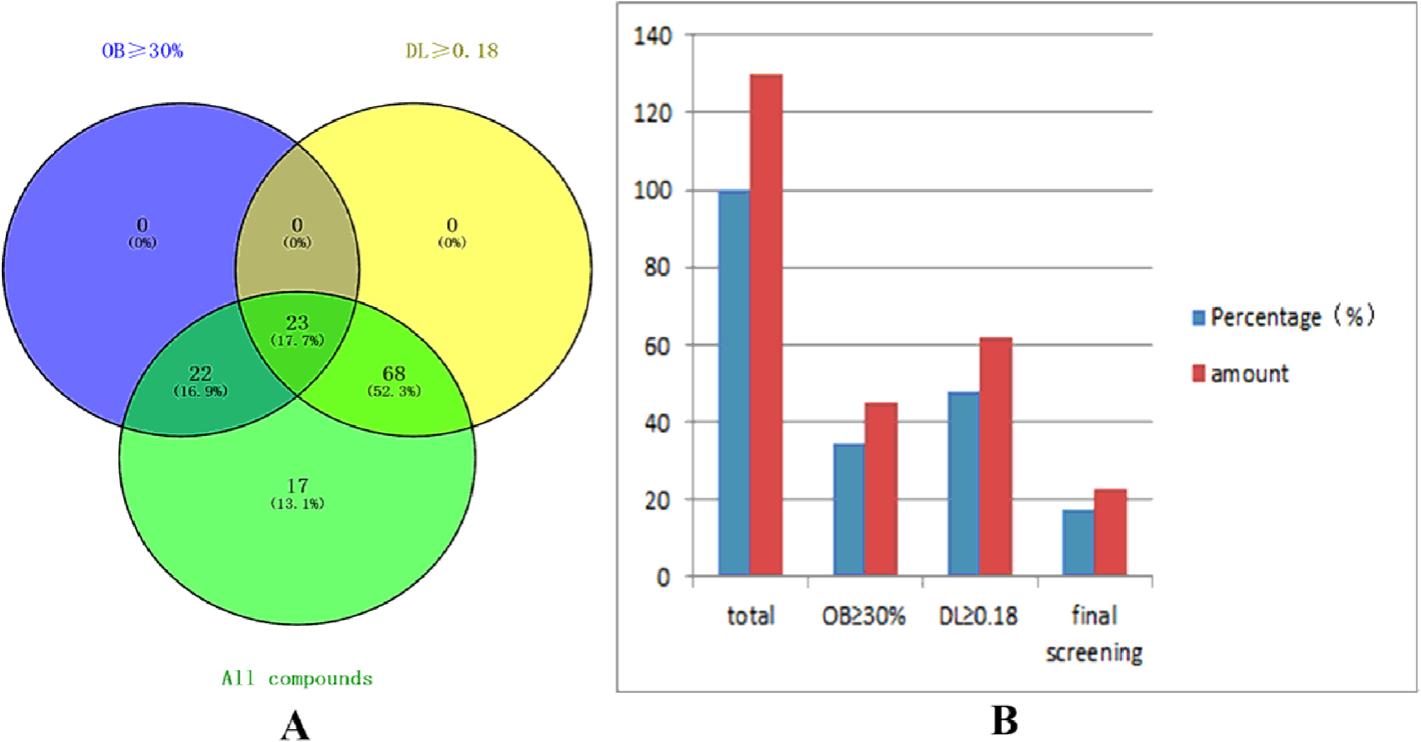
*Epimedium* screening of bioactive compounds. (**A**) Venn diagram: 130 components (green section) and 23 bioactive components screened by two ADME-related models (blue section stands for the components of OB≥30%, yellow section stands for DL≥ 0.18). (**B**) Distributions of components in *Epimedium*

TCMSP database (http://ibts.hkbu.edu.hk/LSPtcmsp.php) is a unique pharmacological platform of TCM system, and it can calculate absorption, distribution, metabolism and excretion (ADME)-related characteristics of natural compounds [22]. A total of 130 components of *Epicedium* were identified from TCMSP. The components were screened with the criteria of OB≥30% and DL≥0.18. A total of 23 bioactive components of *Epicedium* were screened, in which four had no targets. Finally, 19 compounds were collected from TCMSP database (Fig. 1A, B; Table 1).

**Table 1:** Basic information about the active compounds in *Epimedium* (OB≥30%, DL≥0.18)

N.O.	MolId	MolName	OB(%)	DL
1	MOL001645	Linoleyl acetate	42.10	0.20
2	MOL001792	DFV	32.76	0.18
3	MOL003044	Chryseriol	35.85	0.27
4	MOL003542	8-Isopentenyl-kaempferol	38.04	0.39
5	MOL000359	sitosterol	36.91	0.75
6	MOL000422	kaempferol	41.88	0.24
7	MOL004367	olivil	62.23	0.41
8	MOL004373	Anhydroicaritin	45.41	0.44
9	MOL004380	C-Homoerythrinan, 1,6-didehydro-3,15,16-trimethoxy-, (3.beta.)-	39.14	0.49
10	MOL004382	Yinyanghuo A	56.96	0.77
11	MOL004384	Yinyanghuo C	45.67	0.50
12	MOL004388	6-hydroxy-11,12-dimethoxy-2,2-dimethyl-1,8-dioxo-2,3,4,8-tetrahydro-1H-isochromeno[3,4-hisoquinolin-2-ium	60.64	0.66
13	MOL004391	8-(3-methylbut-2-enyl)-2-phenyl-chromone	48.54	0.25
14	MOL004396	1,2-bis(4-hydroxy-3-methoxyphenyl)propan-1,3-diol	52.31	0.22
15	MOL004427	Icariside A7	31.91	0.86
16	MOL000006	luteolin	36.16	0.25
17	MOL000622	Magnograndiolide	63.71	0.19
18	MOL000098	quercetin	46.43	0.28
19	MOL004386	Yinyanghuo E	51.63	0.55

### Depression–*Epicedium* PPI network

**Fig. 2:**
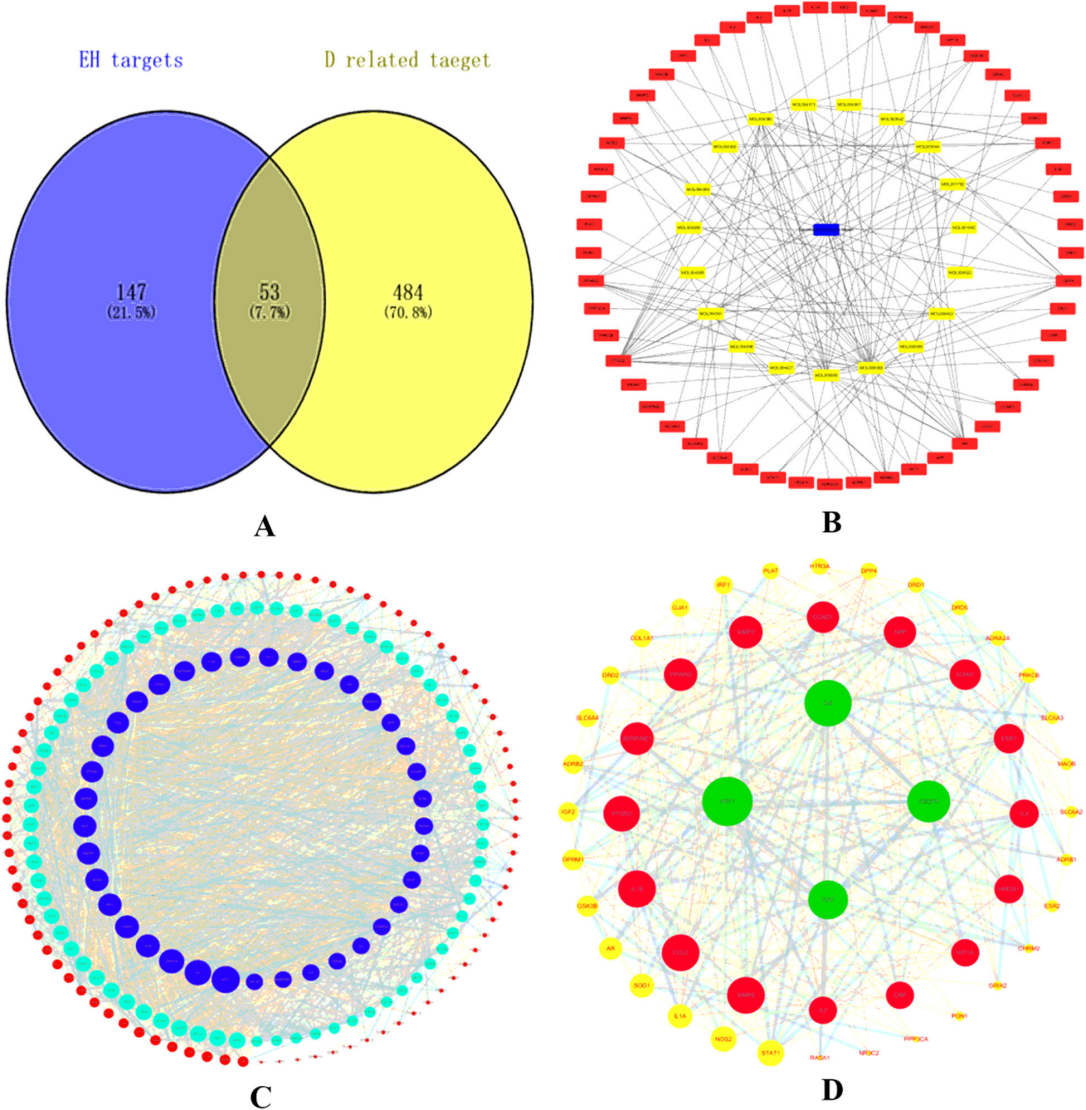
Common-target network. (**A**). Mapping of *Epimedium*- and depression-related targets and the 53 common targets. (**B**). Construction of *Epimedium* component-target visual network. Blue node stands for *Epimedium*, yellow nodes stand for bioactive components, and red nodes stand for predicted targets. (**C**). Interaction network of *Epimedium*-related target proteins. The dark blue nodes represent the big hub nodes. The node size was proportional to the target degree in the network. (**D**). Interaction network of proteins related to the treatment of depression by *Epimedium*. The green nodes represent the big hub nodes. The node size was proportional to the target degree in the network

By using depression as keyword in NCBI database, 537 genes related to depression were retrieved, and 200 target genes of *Epicedium* were collected from the TCMSP database. After intersecting the target genes of *Epicedium* and depression, 53 common genes were found (Fig. 2A). These genes could be the target genes of *Epicedium* in the treatment of depression. Then, we built a visualized *Epicedium*–component–targets network by using Cytoscape 3.6. The nodes of different colors and shapes represented the potential active components and targets of *Epicedium*. The blue nodes represented *Epicedium*, the yellow nodes represented the active components of *Epicedium*, and the red nodes represented the potential antidepressant targets of *Epicedium*. The edges represented the correlation between the active components and the targets (Fig. 2B), confirming the multi-component and multi-target characteristics of *Epicedium*. A total of 199 nodes and 3,302 edges were observed in Fig. 2C, the average node degree was 33.2, and the local clustering coefficient was 0.574 . A total of 53 nodes and 449 edges were observed in Fig. 2D, the average node degree was 16.9, and the local clustering coefficient was 0.662. The size of the node represented the degree value of targets. The larger the node was, the greater the degree value was. The thickness of the edge indicated the combination score. The coarser the edge was, the greater the combination score value was. Data for the degree of each target was shown in Table 2.

**Table 2:** Information about potential antidepressant targets from *Epimedium*

serial number	name	degree	serial number	name	degree
1	AKT1	41	27	OPRM1	15
2	IL6	38	28	IGF2	14
3	VEGFA	34	29	ADRB2	14
4	EGF	31	30	DRD2	13
5	IL1B	29	31	IRF1	13
6	CCL2	29	32	COL1A1	13
7	MMP9	29	33	SLC6A4	13
8	PTGS2	28	34	GJA1	13
9	SERPINE1	25	35	PLAT	12
10	MMP2	25	36	HTR3A	12
11	PPARG	25	37	DPP4	12
12	CCND1	24	38	PRKCB	11
13	APP	24	39	ADRA2A	11
14	ICAM1	23	40	DRD5	11
15	ESR1	22	41	DRD1	11
16	IL4	21	42	SLC6A3	9
17	HMOX1	21	43	ESR2	9
18	HIF1A	20	44	ADRB1	9
19	IL2	20	45	SLC6A2	9
20	CRP	20	46	MAOB	9
21	STAT1	19	47	GRIA2	7
22	NOS2	18	48	CHRM2	7
23	IL1A	17	49	PON1	7
24	SOD1	17	50	PPP3CA	5
25	AR	16	51	RASA1	4
26	GSK3B	15	52	NR3C2	4

### GO analysis of genes targeted by *Epicedium*

**Fig. 3:**
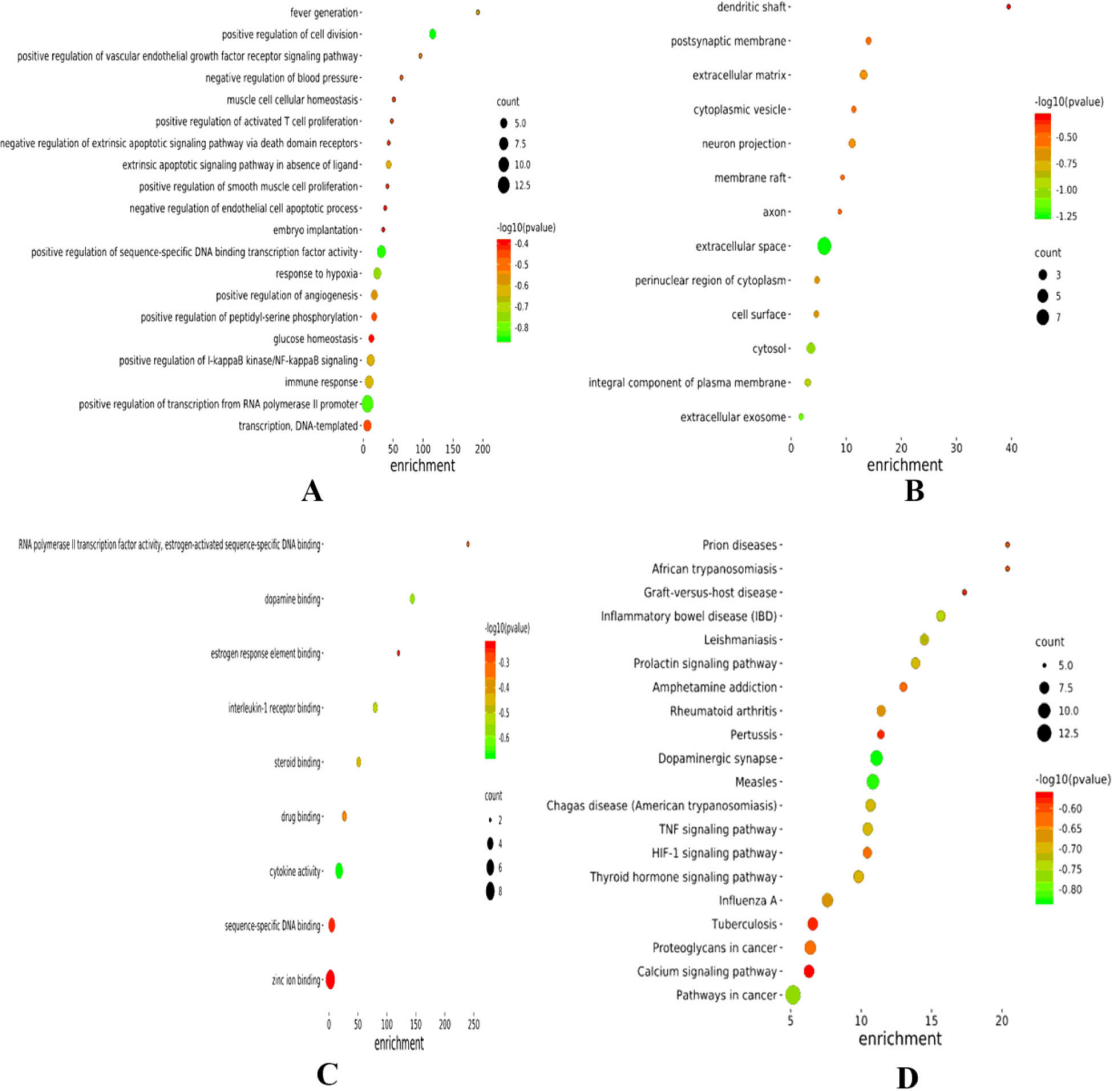
Enrichment of gene ontology (GO) and KEGG pathway of *Epimedium* in the treatment of depression. (**A**).Enriched GO terms for biological process (BP) of potential antidepressant targets from main active ingredients of *Epimedium*. (**B**). Enriched GO terms for the cellular component of potential antidepressant targets from main active ingredients of *Epimedium*.(**C**). Enriched GO terms for molecular function of potential antidepressant targets from main active ingredients of *Epimedium*. (**D**). Enriched KEGG pathways of potential antidepressant targets from main active ingredients of *Epimedium*

David database was applied to analyze the related targets of *Epicedium* for the treatment of depression. In BP analysis, the top ranked targets were distributed in the process of cell division (5 targets/5.9%), positive regulation of sequence-specific DNA binding transcription factor activity (7 targets/8.3%) and transcription from RNA polymerase II promoter (13 targets/15.3%), response to hypoxia (6 targets/7.1%), and immune response (7 targets/8.3%) (Fig. 3A). In CC analysis (Fig. 3B), cell components such as extracellular space (18 targets/21.3%), cytosol (11 targets/13.0%), extracellular matrix (4 targets/4.7%), neuron projection (4 targets/4.7%), integral component of plasma membrane(8 targets/9.4%), and extracellular space(18 targets/21.3%) were at the top. In MF analysis (Fig 3C), molecular functions such as cytokine activity (6 targets/12.8%), dopamine (3 targets/6.4%), IL-1 receptor (3 targets/6.4%), steroid (3 targets/6.4%), and drug binding (3 targets/6.4%), and cytokine activity (6 targets/12.8%) were at the top.

### KEGG analysis of genes encoding proteins targeted by *Epicedium*

David database was used to analyze the KEGG pathway of 53 targets of *Epicedium* for the treatment of depression. According to the p value, the top 20 pathways were selected and shown in Fig. 3D. The color of the nodes in the figure from green to red reflected the p value from large to small. The nodes from small to large reflected the number of related genes in increasing trend. The top ranked pathways were dopaminergic synapse, measles pathways in cancer, inflammatory bowel disease, leishmaniasis, and chagas disease (American trypanosomiasis).

### Molecular docking

**Fig. 4:**
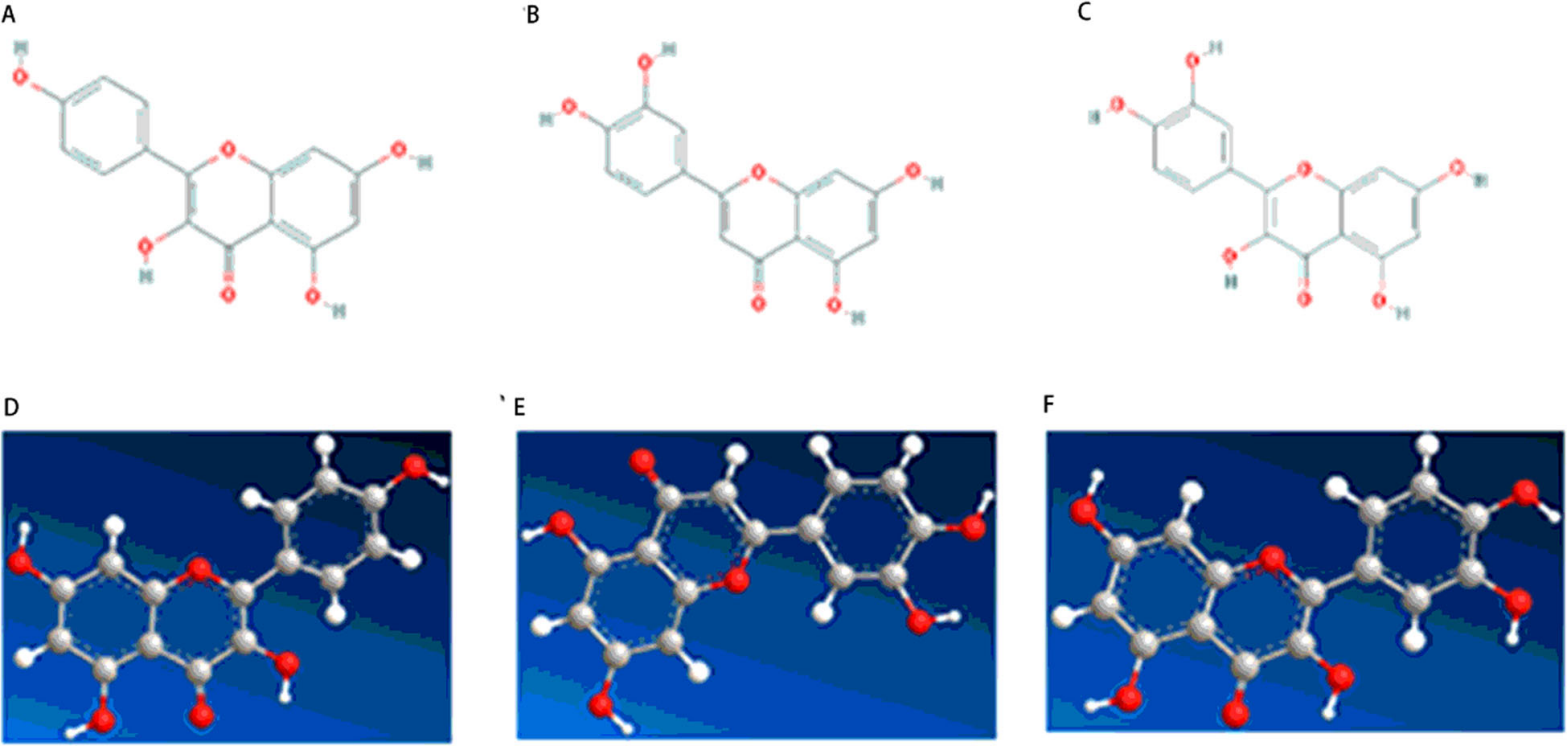
2D and 3D structures of ligands. (**A**) 2D structure of kaempferol. (**B**) 2D structure of luteolin. (**C**) 2D structure of quercetin. (**D**) 3D structure of kaempferol. (**E**) 3D structure of luteolin. (**F**) 3D structure of quercetin

**Fig. 5:**
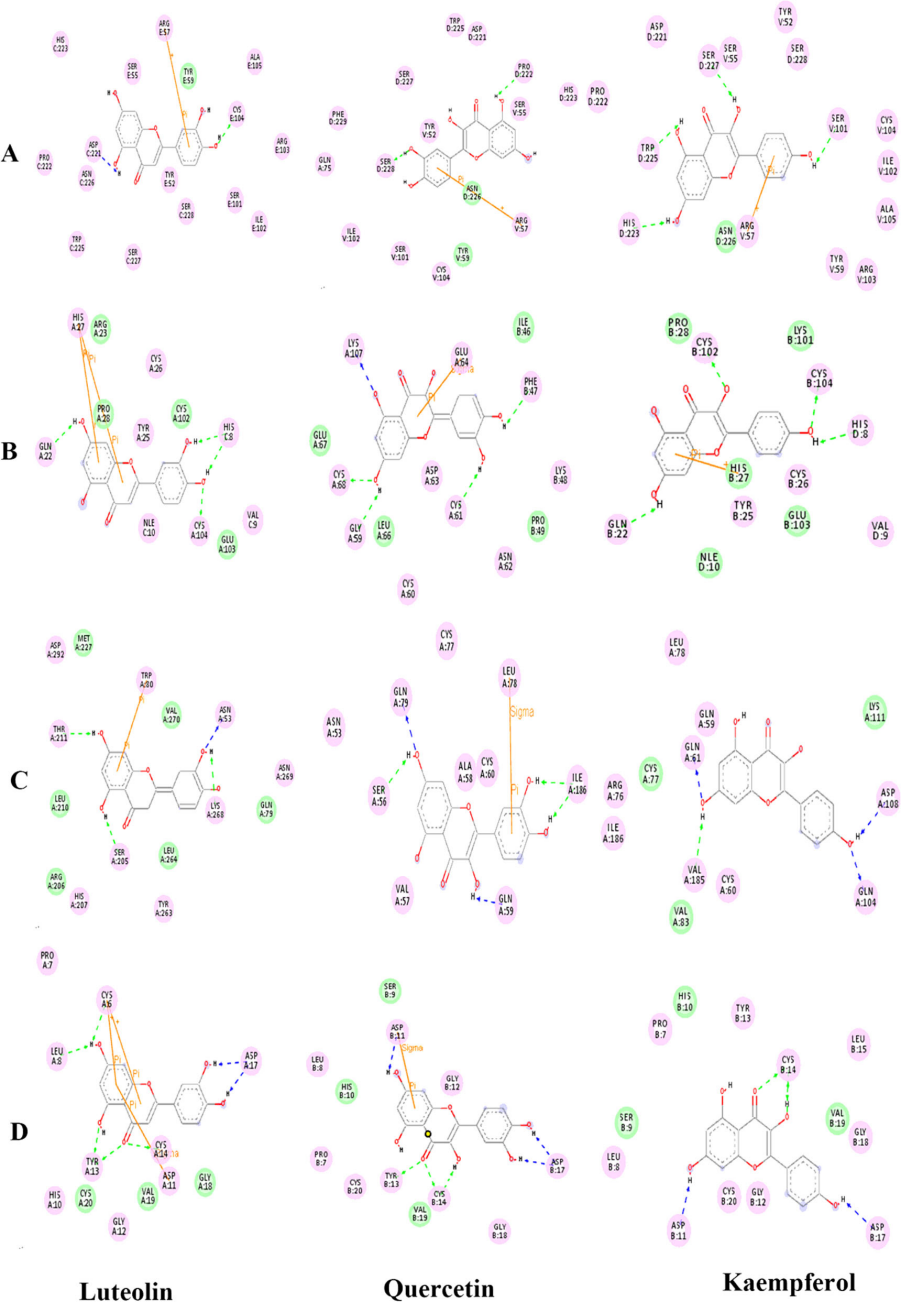
Binding mode of protein and different ligands.(**A**) Binding mode of IL6 with luteolin, quercetin, and kaempferol.(**B**) Binding mode of VEGFA with luteolin, quercetin, and kaempferol. (**C**) Binding mode of AKT1 with luteolin, quercetin, and kaempferol. (**D**) Binding mode of EGF with luteolin, quercetin, and kaempferol. Pink represents the amino acids that form electrostatic force, and green represents the amino acids that form van der Waals force

The results are shown in Fig. 5 and supplementary Figure1-2. The 2D and 3D structures of the ligands are shown in Fig. 4. The estimated free energy of binding and RMSD was summarized in Table 3. The interactions between ligands and target proteins is shown in Table 4. Electrostatic force and van der Waals force are the main forces between ligand and target protein. The binding energies of ligand and receptor were less than -1.19cal / mol, and RMSD were less than 2, indicating that the docking results were good. Fluoxetine was used as positive control, and the binding energies of kaempferol, quercetin and luteolin were lower than that of fluoxetine, indicating that the binding stability of kaempferol, quercetin and luteolin was better than that of fluoxetine.

**Table 3:** Binding energy (kcal/mol) and RMSD of three components of *Epimedium* and target proteins

parameter	Estimated Free Energy of Binding				RMSD			
receptor	IL6	VEGFA	AKT1	EGF	IL6	VEGFA	AKT1	EGF
ligand								
luteolin	-5.48	-6.74	-6.95	-7.33	0.062	0.063	0.067	0.066
quercetin	-5.2	-7.31	-6.95	-6.3	1.165	1.166	1.17	1.17
kaempferol	-5.9	-6.85	-6.82	-6.49	0.064	0.066	0.069	0.069
fluoxetine	-4.77	-5.11	-5.35	-5.71	0.653	0.632	0.643	0.627

**Table 4:** Interaction between ligands and target proteins

Force	electrostatic force				van der Waals force			
receptor	IL6	VEGFA	AKT1	EGF	IL6	VEGFA	AKT1	EGF
ligand								
luteolin	SER C:227、TRP C:225、ASN C:226、ASP C:221、PRO C:222、HIS C:223、SER E:55、ARG E:57、ALA E:105、CYS E:104、ARG E:103、SER E:103、ILE E:102、SER C:228、TYR E:52、TYR E:59	CYS A: 104, HIS C: 8, Val C: 9, TYR A: 25, CYS A: 26, HIS A: 27, GLN A: 22, NLE C: 10	HIS A: 207, TYR A: 263, SER A: 205, LYS A: 268, ASN A: 269, ASN A: 53, TRP A: 80, ASP A: 292, THR A: 211	GLY A: 12, HIS A: 10, ASP A: 11, CYS A: 14, TYR A: 13, LEU A: 8, PRO A: 7, CYS A: 6, ASP A: 17	TYR E: 59	GLU A: 103, CYS A: 102, PRO A: 28, ARG A: 23	GLN A: 79, LEU A: 264, ARG A: 206, LEU A: 210, MET A; 227, VAL A: 210	GYS A: 20, VAL A: 19, GLY A: 18
quercetin	CYS V: 104, ARG V: 57, SER V: 55, HIS D: 223, PRO D: 222, ASP D: 221, TRP D: 225, SER D: 227, TYR V: 52, PHE D: 229, SER D: 228, GLN A: 75, ILE V: 102, SER V: 101	CYS A: 60, ASN A: 62, ASP A: 63, CYS A: 61, LYS B: 48, PHE B: 47, GLU A: 64, LYS A: 107, CYS A: 68, GLY A: 59	GLN A: 59, ILE A: 186, LEU A: 78, ALS A: 58, CYS A: 60, CYS A: 77, GLN A: 79, ASN A: 53, SER A: 56	CYS B: 14, GLY B: 18, ASP B: 17, GLY B: 12, ASP B: 11, LEU B: 8, PRO B: 7, CYS B: 20, TYR B: 13	ASN D: 226, Tyr V: 59	GLU A: 67, LEU a: 66, PRO B: 49, ILE B: 46	/	SER B: 9, VALB: 19, HIS B: 10
kaempferol	ARG V: 57, TYR V: 59, ARG V: 103, ALA V: 105, ILE V: 102, CYS V: 104, SER V: 101, SER D: 228, TYR V: 52, SER V: 55, SER D: 227, ASP D: 221, PRO D: 222, TRP D: 225, HIS D: 223	TYR B: 25, CYS B: 26, VAL D: 9 ,HIS D:8,GLN B:22,CYS B:104,CYS B:102	VAL A: 185, CYS A: 60, GLN A: 104, ASD A: 108, GLN A: 61, LEU A: 78, ARG A: 76, ILE A: 186	ASP B: 11, CYS B: 120, GLY B: 12, ASP B: 17, GLY B: 18, CYSB: 14, LEU B: 15, TYR B: 13, PRO B: 7, LEU B: 8	ASN D: 226	NLE D:10, GLU B :103,HIS B :27, LYS B :101,PRO B:28	VAL A: 83, LYS A: 111, CYS A: 77	SER B: 9, VAL B: 19, HIS B: 10
Fluoxetine(**positive controls**)	ASP B:26, ARG B:30, LYS C:252, ASP C :253, GLU C:23, LYS B :27	CYS B:102, HIS B:27, GLN B:22, CYS B:26	ASN A:31, VAL A:106, THR A:105,ASP A:3	ASP A:17, CYS A:20, CYS A:6,TYR A:13, ASP A :11,CYS A:14, GLY A:18,	SER E :30, ARG C :231, THR E:31, ARG B:182, LEU C :254, TYR E:27	HIS D:8, VAL D:9, PRO B:28, TYR B :25, LYS B :101, ARG B :23, GLU B :103, NLE D:10, CYS B:104,	ASP A:32, VAL A;7, GLY A:109, VAL A:4, LEU A :110	LEU A:15, LEU A:8, VAL A:19, GLY A :12, HIS A :16

"/" means no amino acid residues

## Discussion

Depression is a common mental disorder with a high incidence, recurrence rate, and mortality. Depression not only seriously endangers the lives and health of the people but also causes heavy mental and economic burden to the society, patients, and families. The pathogenesis of depression is very complex. Depression has become a prevalent worldwide health concern. It is still mainly treated with drugs.

*Epicedium* has different effects, such as anti-inflammatory, anti-aging, anti-tumor, anti-oxidation, and anti-inflammatory effects, improves immunization, and acts as antidepressants. In the present study, the main research idea was based on the perspective of disease-target-drug, and we applied the network pharmacology approach to evaluate the anti-depression effects and underlying mechanism of *Epicedium*. These results provide an important reference for the further study of the pathogenesis of depression and the treatment of depression.

By constructing the "drug–target–disease" network, novel drugs and treatment targets could be found [32]. TCMs are difficult to study because of their complex components and huge system. Network pharmacology provides a new idea and perspective for the study of complex TCMs. We integrated information from open databases to predict the interaction between *Epicedium* and its potential protein targets in depression. In the present study, *Epicedium* works in various ways through the effects of multiple compounds and may provide advantages by reducing drug resistance. The results of PPI network show a relationship between the target proteins of *Epicedium*, which was a complex interaction network rather than acting alone. Nineteen active components were obtained by screening, and the three active components with more targets were luteolin (15 targets), quercetin (34 targets), and kaempferol (14 targets). is the results are similar to Naijun Yuan’s results, confirming indicates that flavonoids have obvious antidepressant effect. These flavonoids are common components in many herbal medicines. Their specific roles in the treatment of depression should be clarified [33]. Many studies have demonstrated their neuroprotective effects *in vitro* and *in vivo*. For example, luteolin-regulated genes may act as receptors in the central nervous system to produce antidepressant effects [34, 35]. Quercetin has antioxidant and anti-inflammatory effects in the treatment of neurological diseases. Quercetin alleviates anxiety and depression in chronically stressed rats by protecting neurons from oxidation and inflammation [36]. A study on neuroinflammation induced by lipopolysaccharide in mice indicated that quercetin still can eliminate inflammation by significantly reducing the proliferation of astrocytes in the brain and reducing the expression of inflammatory factors [37]. In addition, quercetin can inhibit neuronal apoptosis by regulating the expression of AKT1 and ASK1/JNK3/Caspase-3, thus preventing anxiety behavior. In the future research, new antidepressant drugs can be developed based on the three compounds.

Previous studies have shown that kaempferol [38], luteolin [39] and quercetin [40] have good antidepressant effects. Quercetin and luteolin have short administration time and low dose, which may be the most impotent for antidepressant property.

The molecular weight of quercetin is 302.24 and its melting point is 314-317 °C, water solubility <0.1g/100 ml at 21 °C. The number of hydrogen bond donors is 5, the number of hydrogen bond acceptors is 7, the number of rotatable chemical bonds is 1, the number of tautomers is 435, the topological molecular polar surface area is 127, the number of heavy atoms is 22, the complexity is 488, and the number of covalent bond units is 1. The kinetic process of quercetin in rats is a two compartment open model with absorption, t1/2(α)=0.19h, t1/2(β)=1.22h, tmax=0.333h [41]. Quercetin was quickly distributed to tissues after it was orally given to rats. The highest concentration of quercetin was observed in stomach in rats, and in order of plasma,liver,kidney,heart,lung,spleen. However, the quercetin concentration in muscle and brain couldn' t be detected by HPLC [42].

The molecular weight of kaempferol is 286.236 and the melting point is 276 °C. The solubility in ethanol is 20 mg/ml. The number of hydrogen bond donors is 4, the number of hydrogen bond acceptors is 6, the number of rotatable chemical bonds is 1, the number of tautomers is 126, the polar surface area of topological molecules is 107, the number of heavy atoms is 21, the complexity is 451, the number of covalent bond units is 1. The pharmacokinetic behavior of kaempferol conformed to the two compartment model, t1/2(α)=0.957±0.172min, t1/2(β)=6.409±1.584. Kaempferol has the characteristics of slow absorption, wide distribution and rapid elimination [43].

The molecular weight of luteolin is 286.236 and the melting point is 330 °C. The number of hydrogen bond donors is 4, the number of hydrogen bond acceptors is 6, the number of rotatable chemical bonds is 1, the number of tautomers is 162, the topological molecular polar surface area is 107, the number of heavy atoms is 21, the surface charge is 0, the complexity is 447, the number of isotopic atoms is 0, the number of covalent bond units is 1, and the solubility is not determined, t1/2(α)= 0.27±0.18h, t1/2(β)= 1.95±0.54h, tmax=0.64± 0.13h [44]. Luteolin and its metabolites preferred to distribute in the gastrointestine, liver, kidney and lung. Biliary excretion dominated the elimination pathways of the conjugated luteolin [45].

The four targets with high degree were AKT1(15), IL6(14), VEGFA(14), and EGF(13). IL-6 is a multi-functional cytokine, which is the basis for various immune responses and acute phase reactions, and it is related to depression and autonomic nervous system symptoms. IL-6 is closely related to hypothalamus pituitary adrenal axis hyperactivity, serotonin metabolism disorder, fatigue, anorexia, depression, and autonomic nervous system symptoms [46]. Akt is a serine/threonine protein kinase, which is an important target of PI3K downstream. Akt has three subtypes (AKT1, AKT2, and Akt3), which play an important role in depression. The activity of Akt protein in the brain tissue of patients with severe depression has been significantly reduced, and Akt can enhance the function of hippocampal stem cells and promote the efficacy of antidepressants [47]. AKT1 has attracted much attention in the study of depression, and it is associated with depression, anxiety symptoms, work, activity, and suicidal tendency of patients with depression [48]. Generally, that AKT1 can regulate the function of antidepressants and contribute to the formation of synaptic plasticity and nerve transmission. Gene-based association identifies loci in VEGFA as potential genetic predictors or SSRI therapeutic response in MDD patients [49]. However, limited studies have been conducted about EGF in depression. The results of the present study show that EGF plays a very important role in the treatment of depression. EGF can be used as another target for the treatment of depression, thus providing a new reference and ideas for the treatment of depression. The possible key components and targets of anti-depression of *Epicedium* are listed above.

The results of molecular docking showed a good binding activity between the three most important components (luteolin, quercetin, and kaempferol) and the four important target proteins (IL6, VEGFA, AKT1, EGF), and the main forms of interaction between components and targets are electrostatic and van der Waals force. Quercetin, kaempferol, and luteolin possess anti-depression effects, indicating that the research method is reasonable and feasible.

Pathway analysis suggested that *Epicedium* may play an antidepressant role by regulating TNF signaling pathway, dopaminergic synapse, and prolactin signaling pathway. TNF, dopamine, calcium signaling pathway, prolactin, and I-kappaB kinase/NF-kappaB signaling play important roles in the pathogenesis of depression [50–52]. As a messenger, Ca^2+^ is involved in the regulation of various neural cell functions, such as the release of neurotransmitters, the construction of cells, and the activation of enzyme system. In the central nervous system, slight changes in Ca^2+^ can lead to significant changes in the function of nerve cells. Therefore, Ca^2+^ balance is an important factor to maintain the structural integrity and normal function of nerve cells. The increase of Ca^2+^ in hippocampal neurons can lead to neuronal apoptosis. Calcium imbalance leads to neuronal apoptosis, then changes of hippocampal structure and function, and eventually leads to depression.

Dopamine, as a monoamine transmitter, is a key neurotransmitter in hypothalamus and pituitary gland. The synthesis, release, reuptake, or metabolic disorders of dopamine can lead to depression. Dopamine deficiency can downregulate the dopamine transporter but upregulate the concentration of D2/3 receptor. The decrease of dopamine transporter in amygdala and the increase of dopamine D2/3 receptor in patients with depression indicate that depression may be related to dopamine deficiency in brain. Hence, dopamine plays an important role in the pathophysiological process of depression. The decrease of dopaminergic neurons and the dysfunction of dopaminergic receptors are risk factors for depression. Tumor necrosis factor has two forms, namely, TNF-α and TNF-β. Its physiological effects are mediated by TNF-R1 and TNF-R2. Serum TNF-α levels in patients with depression significantly increase [53]. In an animal experiment, significant depressive behavior was induced by injecting TNF-α or LPS into the lateral ventricle, Reichenberg [54] found that TNF-α can induce depression and cognitive function changes in humans. In addition, various antidepressants can reduce the level of TNF-α in the peripheral blood of patients with depression. The expression of TNF-α in the dorsolateral prefrontal cortex of patients with severe depression is significantly increased [55]. In addition, Pandey GN found that the expression of TNF-α mRNA and protein in the prefrontal cortex of patients with depression has increased. The serum TNF-α levels can also assess the severity of depression. Therefore, the increase of TNF-α level can lead to the occurrence of depressive symptoms.

Furthermore, more genes were involved in the formation of nerves and synapses. Synapses are the basic structure of information transmission between neurons. They adapt to stimuli by constantly modifying neural connections and circuits. Therefore, synaptic plasticity is the main manifestation of neural plasticity. Depression and other psychological disorders are usually associated with decreased synaptic plasticity in the hippocampus.

Estrogen can affect brain function directly through the estrogen receptor in the brain region, and it plays an important role in depression. It can increase the concentration of 5-hydroxytryptamine, dopamine, norepinephrine, and other neurotransmitters in synapses and affect their release and reuptake. Aged female mice show increased anti-anxiety and -depressant effects after administration of estrogen [56]. In addition, ketamine and its active metabolites have similar affinity to ERα [57]. Ovariectomy can induce depression-like behavior in rats. The implantation of estradiol pellets into both sides of medial amygdala of ovariectomized rats can significantly reduce depressive behavior in rats. This effect may be achieved by activating Erβ, because Erβ agonist can also reverse the depression-like behavior of ovariectomized rats [58]. IL-1 can initiate various immune responses, such as fever, prostaglandin synthesis, neutrophil aggregation and activation, activation of T cells and B cells, and production of cytokines [59]. Animal studies on depression have found that the spleen cell of depression rats induced by chronic mild stimulation have increased the production of IL-1. IL includes 11 factors such as IL-1 α and IL-1 β, among which IL-1α is widely expressed in many kinds of cells and can be activated without any processing [60]. IL-1β is mainly expressed in bone marrow cells. The activation of pattern recognition receptor may be a biological target for innovative treatment of depression. Dahl [59] found that serum IL-1β in patients with depression is significantly higher than that in healthy control group. Huang [60] found that behavioral depression is induced in rats after the intraventricular injection of IL-1 β. The possible mechanism is that IL-1β activates IL-1 receptor type I in hippocampal neural stem cells, which affect nuclear factor kappaB signal transduction pathway, reduce the proliferation of hippocampal cells, and then lead to depression [61]. The loss and overexpression of IL-1β receptor antagonist in the brain of mice can resist the decrease of hippocampal nerve regeneration related to stress, making it less prone to depression [62].

In this study, we applied the method of network pharmcalogy to to explore the antidepressant mechanism of Epimedium for the first time, and docking verification were added to the network pharmacology as complement to predict the drug targets. Protein-protein interaction and KEGG enrichment analysis results show that prolactin signaling pathway and EGF play an important role in the treatment of depression. There are few studies on this aspect. According to the results of this study, we can carry out research on prolactin signaling pathway and EGF in the future depression research.

## Conclusion

System prediction was used to identify *Epicedium* with the potential effect on anti-depression. The network pharmacology, a multi-component and multi-targets analysis fit to the TCM treatment, predicted the potential targets and mechanism of formula, and our previous research shows that *Epimedium* extract has good antidepressant effect. This research indicated that prolactin signaling pathway and EGF play an important role in the treatment of depression. The follow-up study could discuss through the regulation of prolactin signaling pathway and EGF in the treatment of depression , which must be thoroughly tested *in vivo and in vitro*.

## Supplementary Information

**Additional file 1: Supplementary Figure 1**. The four targets were IL6 (PDB ID: 5fuc), VEGFA (PDB ID: 6d3o), AKT1(PDB ID: 6s9x), and EGF (PDB ID:1jl9). The top three compounds were leteolin, quercetin, and kaempferol. The binding energies of ligand compounds and protein receptors were as follows. The binding energy ranges from −7.33 kcal/mol to − 5.20 kcal/mol. Structural model of active ingredients with hub targets. (**A**) Structural model of IL6 with luteolin, quercetin, and kaempferol. (**B**) Structural model of VEGFA with luteolin, quercetin, and kaempferol.(**C**) Structural model of AKT1 with luteolin, quercetin, and kaempferol. (**D**) Structural model of EGF with luteolin, quercetin, and kaempferol. **Supplementary Figure 2**. Binding site of active ingredients with hub targets.(**A**) Binding site of IL6 with luteolin, quercetin, and kaempferol.(**B**) Binding site of VEGFA with luteolin, quercetin, and kaempferol. (**C**) Binding site of AKT1 with luteolin, quercetin, and kaempferol. (**D**) Binding site of EGF with luteolin, quercetin, and kaempferol.

## Data Availability

The datasets used and/or analysed during the current study are available from the corresponding author on reasonable request.

## Abbreviations

PPI: protein-protein interaction
GO: Gene Ontology
KEGG: Kyoto Encyclopedia of Genes and Genomes
SPT: Sucrose Preference Test
FST: Forced Swimming Test
TCMSP: Traditional Chinese Medicine Systems Pharmacology
LPS: Lipopolysaccharide
MDD: Major Depressive Disorder
EGF: Epidermal Growth Factor
VEGFA: Vascular endothelial growth factor A
IL6: Interleukin-6
AKT1: RAC-alpha serine/threonine-protein kinase
