# Supplementary material for: Molecular mechanism of Epicedium treatment for depression based on network pharmacology and molecular docking technology
Source: BMC Complement Med Ther. 2021 Sep 3;21:222. doi: 10.1186/s12906-021-03389-w (PMC8417989; doi:10.1186/s12906-021-03389-w)

Supplementary Figure 1. The four targets were IL6 (PDB ID: 5fuc), VEGFA (PDB ID: 6d3o), AKT1(PDB ID: 6s9x), and EGF (PDB ID:1jl9). The top three compounds were leteolin, quercetin, and kaempferol. The binding energies of ligand compounds and protein receptors were as follows. The binding energy ranges from −7.33 kcal/mol to − 5.20 kcal/mol. Structural model of active ingredients with hub targets. (A) Structural model of IL6 with luteolin, quercetin, and kaempferol. (B) Structural model of VEGFA with luteolin, quercetin, and kaempferol.(C) Structural model of AKT1 with luteolin, quercetin, and kaempferol. (D) Structural model of EGF with luteolin, quercetin, and kaempferol.


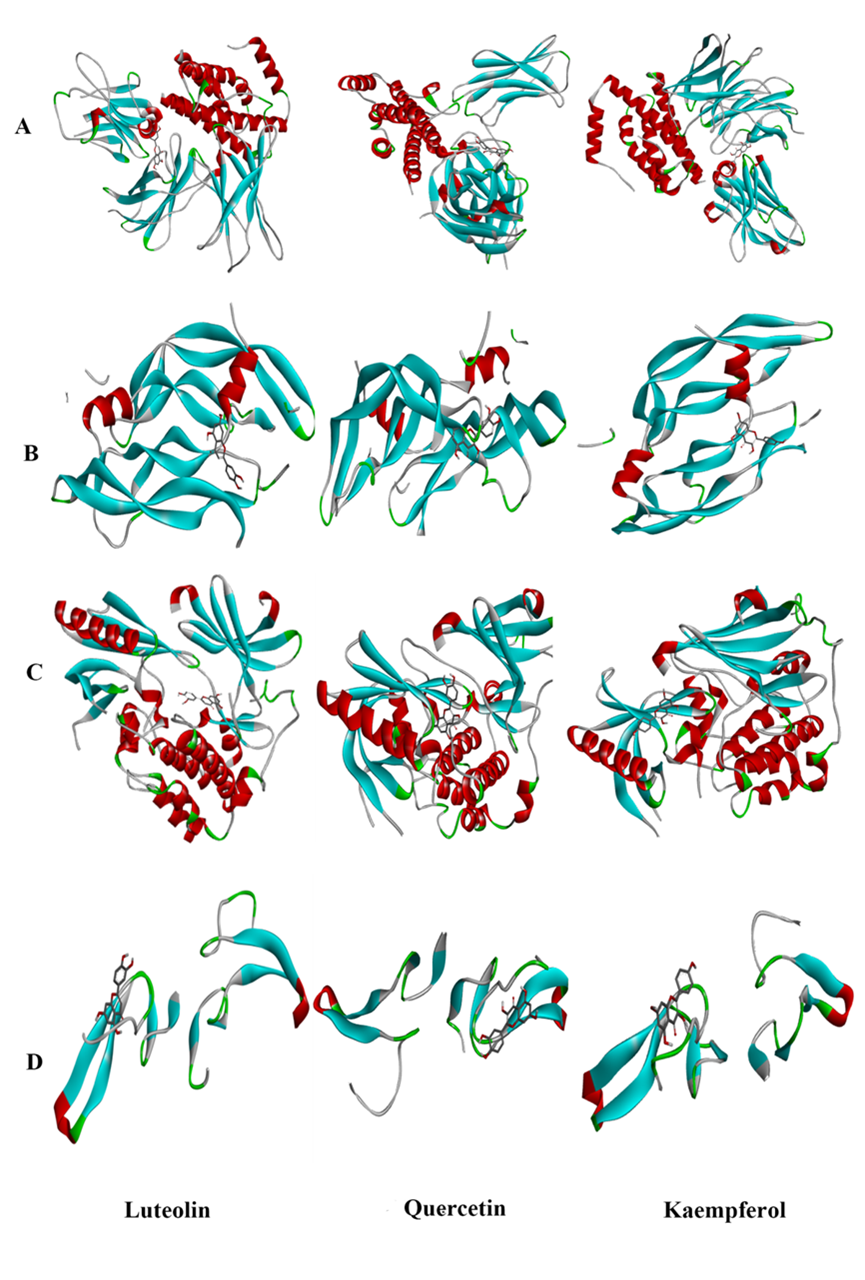


Supplementary Figure 2. Binding site of active ingredients with hub targets.(A) Binding site of IL6 with luteolin, quercetin, and kaempferol.(B) Binding site of VEGFA with luteolin, quercetin, and kaempferol. (C) Binding site of AKT1 with luteolin, quercetin, and kaempferol. (D) Binding site of EGF with luteolin, quercetin, and kaempferol.


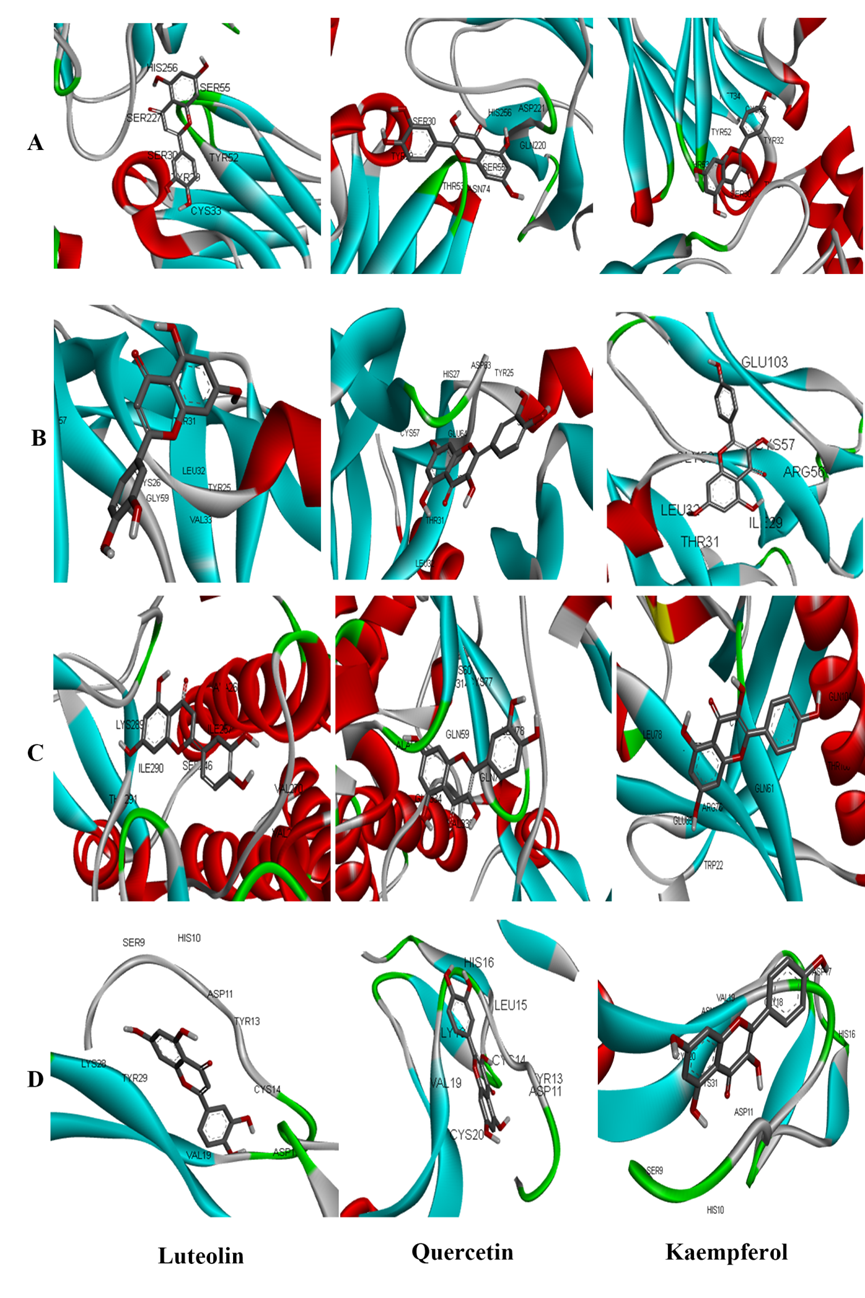

Supplement: Supplementary file 1 — Additional file 1: Supplementary Figure 1. The four targets were IL6 (PDB ID: 5fuc), VEGFA (PDB ID: 6d3o), AKT1(PDB ID: 6s9x), and EGF (PDB ID:1jl9). The top three compounds were leteolin, quercetin, and kaempferol. The binding energies of ligand compounds and protein receptors were as follows. The binding energy ranges from −7.33 kcal/mol to − 5.20 kcal/mol. Structural model of active ingredients with hub targets. (A) Structural model of IL6 with luteolin, quercetin, and kaempferol. (B) Structural model of VEGFA with luteolin, quercetin, and kaempferol.(C) Structural model of AKT1 with luteolin, quercetin, and kaempferol. (D) Structural model of EGF with luteolin, quercetin, and kaempferol. Supplementary Figure 2. Binding site of active ingredients with hub targets.(A) Binding site of IL6 with luteolin, quercetin, and kaempferol.(B) Binding site of VEGFA with luteolin, quercetin, and kaempferol. (C) Binding site of AKT1 with luteolin, quercetin, and kaempferol. (D) Binding site of EGF with luteolin, quercetin, and kaempferol. [file 12906_2021_3389_MOESM1_ESM.docx]
